# Protecting the patches from the footprints: examining the land use factors associated with forest patches in Atewa range forest reserve

**DOI:** 10.1186/s12862-021-01758-0

**Published:** 2021-02-15

**Authors:** Williams Agyemang-Duah, Joseph Oduro Appiah, Dina Adei

**Affiliations:** 1 Department of Planning, Kwame Nkrumah University of Science and Technology, Private Mail Bag, Kumasi, Ghana; 2 School of Environmental Planning, University of Northern British Columbia, 3333 University Way, Prince George, BC V2N 4Z9 Canada

**Keywords:** Forest patches, Land use, Forest reserve, Forest fragmentation, Predictive model, Logistic regression analysis

## Abstract

**Background:**

Land use practices are noted to contribute to changes in forest landscape composition. However, whereas studies have reported the intermix of land uses and forest patches and measured the direct impacts of land uses on forest patches, little is known regarding the spatially-explicit association between the most recent forest patches and land use footprints in protected areas. In this study, we use methods from GIS, remote sensing, and statistics to model the spatial relationship between footprints of land uses and patches of forest cover by drawing on geospatial data from the Atewa range forest reserve (ARFR).

**Results:**

The study finds that forest patches that are within 1 km from agricultural land use footprints (AOR = 86.625, C.I. 18.057–415.563, *P* = 0.000), logging sites (AOR = 55.909, C.I. 12.032–259.804, *P* = 0.000), mine sites (53.571, C.I. 11.287–254.255, *P* = 0.000), access roads (AOR = 24.169, C.I. 5.544–105.357, *P* = 0.000), and human settlement footprints (AOR = 7.172, C.I. 1.969–26.128, *P* = 0.003) are significantly more likely to be less than the mean patch area (375,431.87 m^2^ = 37.54 ha) of forest cover. A ROC statistic of 0.995 achieved in this study suggests a high predictive power of the proposed model.

**Conclusion:**

The study findings suggest that to ensure sustainable land uses and ecological integrity, there is a need for land use policies and land management strategies that ensure responsible livelihood activities as well as further restrictions on logging and mining in the globally significant biodiversity area.

## Background

Studies in sub-Saharan Africa as well as those with global focus have noted that anthropogenic land use continues to threaten forest resources despite efforts being made to ensure sustainable land uses in locations where communities are connected to land for their livelihoods. Indiscriminate land use in forest areas is likely to accelerate forest cover loss, forest patch fragmentation, loss of ecosystem services, and land degradation [1–4]. Thus, from a broader perspective, these land use impacts on forest resources are indirectly connected to the malfunctioning of the forest ecosystem and contribute to global environmental and climate change [5, 6]. Hence, reducing forest loss and fragmentation has been one of the answers to global climate change [7, 8].

Recent studies have identified several anthropogenic land uses that contribute to forest loss, forest fragmentation and land degradation [9, 10]. Agriculture has been identified as one of the factors contributing to forest change, especially in the tropical regions of the world [11–16]. Related to agricultural activities is logging, which also involves the removal of forest cover [17–19]. In recent times, the rush for gold and other mineral resources by local communities and multinational corporations has made mining activities a major contributor to the recent forest cover loss and fragmentation [20–24]. Additionally, urban expansion, including human settlement development and expansion in road networks, also contributes to forest cover loss and fragmentation [22, 25]. However, the land use types and their legacies in different locations are likely to be influenced by local, national and international dimensions and thus, it would be necessary to study land use legacies through the lens of socio-economic, spatial, and environmental conditions in many locations.

The Atewa Range Forest Reserve (ARFR) was established and designated as a forest reserve in 1926 and has also been allocated as a Globally Significant Biodiversity Area (GSBA) and an Important Bird Area (IBA) [26]. In spite of being a protected area and GSBA, the ARFR is a hotspot of anthropogenic activities [27–29]. For instance, according to the Ministry of Lands and Natural Resources of Ghana [30], the ARFR is seriously being threatened by open cast mining and illegal mining (locally called ‘galamsey’). Thus, there is a likelihood that the land uses would contribute to forest cover loss, forest patch fragmentation and land degradation.

Studies in forest ecosystems, especially in the tropical forest reserves have mostly focused on forest cover change from land uses [28, 31–36]. However, none of these studies have downscaled their units of measurement to patch-mosaic level and modelled the association between land uses and patches of forest, especially in locations where agriculture is a major land use activity. We fill this knowledge gap by modelling the likelihood of the occurrence of patches of forest that are less than the mean patch area within a given distance from the land use hotspots using land use and forest patch spatial data from the ARFR. The study aims at presenting a model that predicts the association between land uses and patch size by incorporating the spatial dimensions of land uses and patches of forest. We hypothesise that forest cover patches are not likely to be less than the mean patch area even if they are within 1 km from land use footprints. This hypothesis is built from previous studies [37, 38], which indicate that 70% of the world’s forests are within 1 km of forest edge. Moreover, most of the forest cover patches are well within the range which is made up of different human activities, altered microclimate, and nonforest species that are likely to influence and degrade forest ecosystems [37, 38].

We use a state-of-the-art random forest (RF) machine learning classification algorithm for processing a Landsat image into land use and land cover categories. Moreover, we combine the Landsat image with land use data from a high-resolution aerial image to reduce the effects of scale on the study results. Hence, we present methods from geographic information systems (GIS), remote sensing and statistics to process geospatial data and show the relationship between land uses and forest cover patches. A similar approach has been used successfully in a study to model forest fragmentation, land use and other factors in northeastern China [39]. This study presents important information that reflects environmental, social and economic conditions and directly informs forest policies and management plans about the need to protect forest resources and ensure sustainable land uses in and around protected landscapes.

## Results

The study shows that most of the forest patches are not within 1 km of the different land use footprints (see Table 1). For instance, the study outcome shows that 756 forest patches are not within 1 km of the agricultural footprints in the ARFR. Similarly, 723 of the forest patches are not found within 1 km as compared to 142 of them that are within 1 km from the logging footprint.

**Table 1:** Categorical variables coding and frequencies

Independent variables	Frequency	Parameter coding
Patches within 1 km from human settlement		
No	758	.000
Yes	107	1.000
Patches within 1 km from access roads		
No	751	.000
Yes	114	1.000
Patches within 1 km from mine sites		
No	723	.000
Yes	142	1.000
Patches within 1 km from logging sites		
No	754	.000
Yes	111	1.000
Patches within 1 km from agricultural land		
No	756	.000
Yes	109	1.000

The outcome of the multivariate analysis is as follows (also see Table 2). First, the analysis reveals that forest patches that are within 1 km from agricultural land are about 86.625 times significantly more likely to be less than the MPA of 375,431.87 m^2^ (37.54 ha). Second, the outcome of the analysis shows that forest patches that are within 1 km from access roads are about 24.169 times significantly more likely to be less than the MPA. Moreover, the analysis shows that forest patches that are within 1 km from the mine site footprints are about 53.571 times significantly more likely to be less than the MPA. Also, the study finds that patches that are within 1 km from the logging site footprints are about 55.909 times significantly more likely to be less than the MPA. Furthermore, the study finds that forest patches that are within 1 km from the human settlement footprints are about 7.172 times significantly more likely to be less than the MPA of forest patches in the ARFR. With this study outcome regarding the relationship between the area of forest cover patches and land uses, we reject the null hypothesis that forest cover patches are not likely to be less than the MPA even if they are within 1 km from land footprints. The standard errors (SEs) (see Table 2) for these estimates of likelihoods range between 0.660 and 0.800.

**Table 2:** Results of analysis showing the relationship between the dependent and independent variables

Independent variable	B	S.E	Sig	AOR	95% C.I. for AOR	
					Lower	Upper
Patches within 1 km from AL (No)				1 (*ref*)		
Patches within 1 km from AL (Yes)	4.462	.800	.000**	86.625	18.057	415.563
Patches within 1 km from AR (No)				1 (*ref*)		
Patches within 1 km from AR (Yes)	3.185	.751	.000**	24.169	5.544	105.357
Patches within 1 km from MS (No)				1 (*ref*)		
Patches within 1 km from MS (Yes)	3.981	.795	.000**	53.571	11.287	254.255
Patches within 1 km from LS (No)				1 (*ref*)		
Patches within 1 km from LS (Yes)	4.024	.784	.000**	55.909	12.032	259.804
Patches within 1 km from HS (No)				1 (*ref*)		
Patches within 1 km from HS (Yes)	1.970	.660	.003**	7.172	1.969	26.128
Constant	− 8.204	1.288	.000	12,304.784		

*AL* agricultural land, *AR* access roads, *MS* mine site, *LS* logging site, *HS* human settlement, *B* coefficients of the models, *S.E.* standard error, *AOR* adjusted odds ratio, *CI* confidence interval

**Relationship is statistically significant if P < 0.05 at a 95% confidence interval

### Model goodness-of-fit and model robustness

**Fig. 1:**
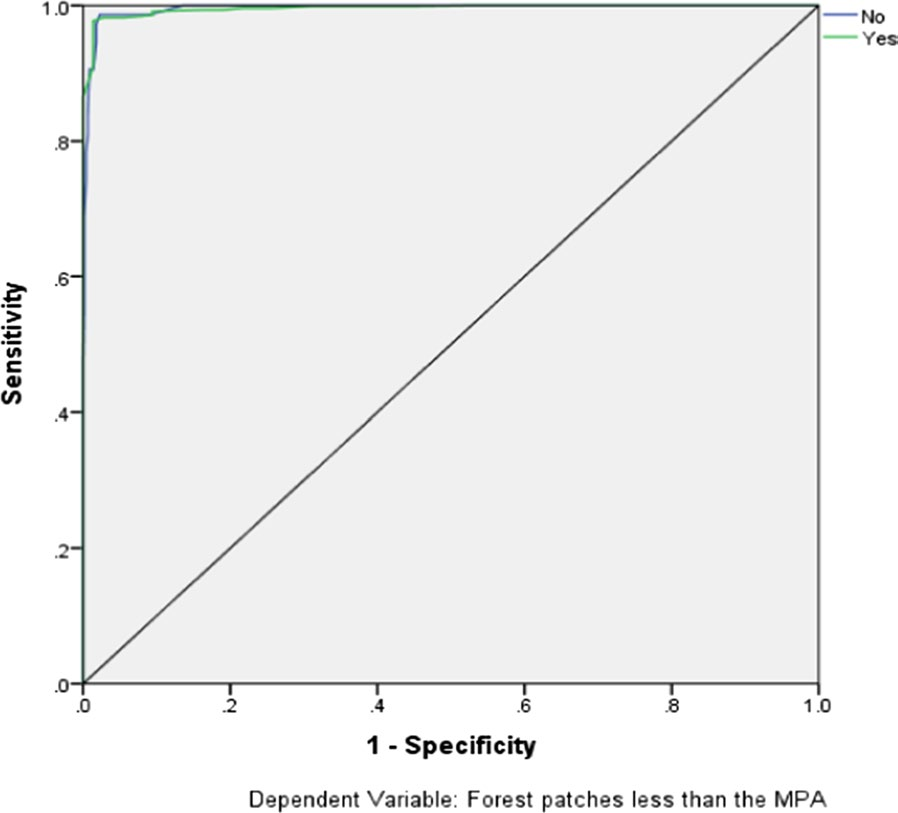
The receiver operating characteristics (ROC) curve showing the predictive capability of the model (*MPA* mean patch area of forest cover). This figure was created by authors during the statistical analysis of the relationship between the area of forest cover patches and land uses

The robustness of the model has been measured and the results are as follows. Firstly, given the set of data, the outcome (*P* > 0.05) of the Hosmer and Lemeshow test (see Table 3) shows that the model is a good fit to the data. Secondly, the Omnibus Tests of Model Coefficients show a significant difference between the based model (without explanatory variables) and the current model with explanatory variables (*P* < 0.05). Thirdly, the ROC curve shows that there is a near-perfect prediction of 0.995 (see Fig. 1). Moreover, the overall accuracy for the prediction of patches that are less than the MPA and that of those that are less than the predicted accuracy is 90.5% and 99.2%, respectively (see Table 4). Furthermore, the pseudo R^2^ (Nagelkerke R Square) shows that the independent variables explain about 89.3% of the variation in the model’s dependent variable.

**Table 3:** Omnibus tests of model coefficients

	Chi-square	df	Sig
Step	435.161	5	.000***
Block	435.161	5	.000***
Model	435.161	5	.000***

***For the omnibus tests of model coefficient, P < 0.05, and, hence results are statistically significant

**Table 4:** Summary of the observed and predicted outcome

Observed	Predicted		
	Less than MPA		Percentage correct
	No	Yes	
Less than MPA			
No	785	6	99.2
Yes	7	67	90.5
Overall percentage			98.5

MPA is the mean patch area

## Discussion

This study examines the association between spatially-explicit land use factors and the most recent forest patches on the landscape. We present a model that shows a significant relationship between the forest patches and the land use factors. Whereas relative measures have been used to show the robustness of similar models in previous studies [40–42], we used the area under the curve or the ROC and other measures to determine the robustness of the model. The ROC statistic of 0.995 suggests a near-perfect model fit [43], and thus, the resultant model achieved in the modelling process is not a result of a random chance. However, the use of a different distance threshold of a spatial relationship between land use types and forest cover patches in other forest areas would likely produce a different ROC statistic. Similar to the ROC statistic result, other measures such as the Omnibus Tests of Model Coefficients, the Hosmer and Lemeshow test, and the R^2^ indicate that the model is robust and thus the model fits the dataset used in the analysis. The uncertainties in the classification of the forest cover patches using the RF machine learning algorithm would likely impact the results. For instance, the size of the forest patches would likely be overestimated or underestimated due to the imperfections associated with the current state-of-the-art machine learning classification algorithm. Specific spatially-explicit land use variables and their relationship with the mean patch area have been discussed as follows.

The spatially-explicit model demonstrates significantly different levels of associations between land uses and recent patches of forest cover. All the spatially-explicit land use factors are significantly related to the forest patch sizes and thus, adequately explain the area of forest patches in the ARFR. Generally, the study findings suggest that forest patches are likely to be smaller than the mean patch area if they are found near the land use footprints, implying that forest patches farther away from land use footprints are less likely to be smaller than the mean patch area of patches on the landscape. That is, the part of the forest patches, especially at the fringes of the reserve where human influences are high are more likely to have patches less than the mean patch area. This study outcome supports the assertion [44] and findings from KwaZulu–Natal midlands-South Africa [45], Midwest of the United States [46] that human-dominated landscapes are more likely to have smaller patches.

It has been argued that in the human-dominated landscapes (e.g., the corn belt of the midwestern United States, rural landscapes in Africa and the Latin American Amazonia), where human activities and impacts on the environment are on the rise, there is an opportunity to preserve aspects of the landscape pattern and ensure sustainable land uses that take care of the livelihoods of the present as well as future generations [46–48]. In the ARFR where farmer encroachment along the forest reserve fringes is on the rise [28], better land preservation measures will be needed. Thus, in the ARFR, the challenge will be about how to maintain land uses and still maintain the protected area status of the reserve. However, lessons can be learnt from other locations where a better reconciliation between land uses and environmental protection has resulted in a ‘win–win’ situation. For instance, in the Wolong Nature Reserve in Sichuan province of China, where 90% of the local inhabitants are farmers, there has been a better reconciliation of ecological and socio-economic objectives by providing non-agricultural employment opportunities for local populations to ensure improvement in local livelihoods [49]. Consequently, this management strategy around the reserve has reduced anthropogenic pressure on the reserve, specifically, through reduced fuelwood collection and agricultural activities [49].

As expected in the ARFR, an area surrounded by an agrarian landscape, agricultural footprints are the most relevant factor influencing the area of forest patches. In relating the footprints of agricultural activities to the patches of forest, the outcome of the analysis implies that the forest patches area would likely be larger if they are found beyond 1 km from the agricultural footprints. Thus, this study finding suggests that forest patches that are closer to the farmlands are more likely to be smaller as compared to the patches that are farther away from the farmlands. This study outcome is related to the finding from previous studies in KwaZulu–Natal midlands, South Africa and the Eastern North American agricultural landscape that assert that cropland and pasture appear to impede the expansion of forest patches [45, 50]. The onus is on land managers to meet the ever-increasing demand for agricultural land for food production without compromising the biodiversity and many ecosystem services provided by forests. For instance, a previous study in the ARFR in Ghana has noted that the ARFR serves as a watershed for important river systems, namely, the Densu, Ayensu, and Birim [51]. Hence, the continuous indiscriminate encroachment by farmers at the forest reserve fringes would likely threaten the sustainability of the rivers and the services they provide for the surrounding communities that depend on these river systems for water. Land managers could ensure socio-ecological sustainability by allowing farming activities provided farmers are willing to mix their crops with tree seedlings meant to reforest the degraded patches of forest cover.

Additionally, the study finds that forest patches that are within 1 km from logging sites are significantly more likely to be less than the mean patch area of forest. This implies that those patches that are not closer to the logging sites are likely to be larger as compared to those that are closer to the logging sites. As indicated in the odds ratios of the model, logging sites appear to be the second most important factor to associate with the patches. Generally, logging activities in forest landscapes produced patterns characterized by high patch and edge densities and small patch areas [44, 52]. For instance, in a related study in Indonesia, Gabon, Democratic Republic of Congo, Republic of Congo, Suriname, and Mexico, Putz et al. [53] found that the proportion of intact forest decreases with an increase in the harvest in the access portions of logging blocks. Thus, based on this finding and that of our study, forest management activities could ensure that logging activities are not overly dispersed across the landscape to contribute to patch area reduction and subsequent fragmentation. Commercial logging activities should be done taking into consideration the spatial context of spreading forest patch fragmentation and land degradation.

Forest patches, from the multivariate analysis, are more likely to be less than the MPA if they are found within proximity to mine sites in the ARFR. This finding implies that forest patches would likely be larger when they are far away from the mine sites as compared to patches that are close to the mine sites. Whereas not directly related to the findings of this study, previous studies in forested landscapes in East Jaintia Hills-India, Brazilian Atlantic Forest and ridgetop biota of Appalachian forests have shown that reduced forest cover is mostly associated with mine sites [54–56] and thus, it is likely that forest patches are reduced or lost as a result of mining activities in such landscapes. Also, clearing of forest for mining activities would likely create room for other activities (e.g., road construction and human settlement development) and access to other parts of the forest cover. Hence, we argue that there might be other factors accounting for the forest patch area on the landscape and there is the need to always consider multiple variables in determining the forest patch pattern. However, such consideration is dependent on the availability of data for the variables.

Moreover, the study outcome shows that forest patches that are within 1 km from the access roads are more likely to be less than the MPA. This study outcome suggests that the forest patches would likely be larger in an area far away from the access roads as compared to patches that are closer to the access roads. In a similar study in northeastern China [39], it was found that forest patches that are closer to roads (highways) are more likely to be smaller as compared to the patches that are far away from the roads. Moreover, the outcome of our study corroborates a related study from tropical forest environments (Indonesia, Gabon, Democratic Republic of Congo, Republic of Congo, Suriname, and Mexico) which shows that more forests are left intact in areas farther from roads away as compared to forest patches that are within proximity to roads [53]. Global forest landscape analysis and studies from Addis Ababa-Ethiopia and eastern Germany have used large patch size as a measure of forest intactness, ecological integrity, and ecosystem functioning [57–59]. Thus, in the ARFR, forest patches would likely be of high ecological integrity if they are large and not within 1 km from access roads. With that being implied, access roads, are also likely to facilitate other land uses and contribute to forest loss [60]. For instance, Sahana et al. [61] in a study in the Song Watershed in India have found that human intervention along access roads has contributed to forest degradation, one of the resultant attributes of forest fragmentation. However, Kaczan [62] in a study in India found that access road development contributes to forest transition (both losses and gains). Therefore, there should be management initiatives and policies to harness the positive aspect of access roads while mitigating impacts to ensure long term sustainable land uses.

Furthermore, from the study, it is observed that forest patches that are within 1 km from human settlements are more likely to be less than the MPA of forest patches on the landscape. This study result suggests that it is more likely that smaller patches would be found near footprints of human settlements. In a related study from northeastern China, it was found that forest cover patches reduced with distance to human abodes, including cities [39]. Bar-Massada, Radeloff, and Stewart [63] in a review of literature on studies from different locations (e.g., South Africa, NewZealand, and the United States) identified human settlement interface and intermix with wilderness as a mode of forest habitat fragmentation, including reduced forest fragments for habitats and ecosystem processes. Also, Sahana et al. [61] have found that demand for land for human settlement development reduces the amount of forests and contributes to the formation of a mix of forest patches and land uses. Thus, the natural area-land use interface is likely to contribute to the creation of smaller patches of forests. Since human settlement development facilitates other land uses (e.g., agriculture), forest management strategies and policies should be multidimensional in outlook, encompassing many proactive and ad-hoc ways of managing the factors that are likely to influence the forest cover patches in protected areas.

The results from this study would likely be applicable for forest and land use management purposes in other locations, especially in tropical forests and other forest areas in Ghana and beyond where anthropogenic activities such as logging, mining, agricultural activities, creation of access roads, human settlement development are on the rise for socio-economic reasons. For instance, in the Latin American Amazon, East African, and South Africa forest areas, the land use characteristics are similar to that of ARFR. These forest areas are characterised by activities such as logging, mining, and agricultural activities [24, 64–67]. Thus, in the light of these human activities, climate change concerns and the findings from our study in the ARFR, forest cover patches in the tropical forests and other forest regions would need protection to preserve biodiversity and ensure sustainable land uses.

## Conclusion

Our study aimed at modelling land uses and the area of patches that are less than the mean patch area of the forest using remote sensing and GIS data from the Atewa Range Forest Reserve. We provide a model that explains the relationship between land uses and forest cover patches, and thus we advance previous studies that focused mainly on how land uses contribute to the fragmentation of the forest cover patches. The study finds that the forest patches are within 1 km from the agricultural land are significantly more likely to be less than the mean patch area of forest cover. This study outcome is expected in a protected area surrounded by agrarian landscapes where previous studies have reported encroachment due to expansion in agricultural land. Moreover, the study finds that patches of forest that are within 1 km from the logging site are significantly more likely to be less than the mean patch area of forest. Furthermore, we find that forest patches that are within 1 km from mine sites are significantly more likely to be less than the mean patch area of forest cover. The proposed model is deemed fit and that the variables in the models could be adjusted for different locations to reflect biophysical and socio-economic characteristics.

The outcome of the study suggests that forest patches that are within the 1 km from spatially-explicit land use factors, as specified in the model, are more likely to be less than the mean patch area of forest cover. However, this study outcome can differ in other landscapes depending on the biophysical characteristics of the land and the prevalent land uses. Thus, this presents unique land management problems in different locations depending on the socio-economic characteristics. Despite the need for proactive location-specific land management programmes to address the growing pressure on forest patches, the basic tenets of the programmes should focus on the need to reconcile the socio-economic needs of local communities with the ecological needs of the forest ecosystem. Furthermore, land use and forest management programmes should be supported by a robust landscape pattern assessment overtime to provide insights into the rate at which the forest landscape quality is deteriorating or improving. For instance, landscape pattern assessment could include the use of widely accepted landscape measurement approaches [68, 69], which have been used in previous studies e.g., [70–74].

Even though choosing a distance threshold for the land uses was based on extensive analysis from different continents, an adjustment of the distance threshold could change the study results. Thus, choosing a distance threshold is likely to be arbitrary and can bias the study results if it is not theoretically grounded in the literature. However, continuously testing the 1 km threshold in different locations would likely help to determine its robustness and ensure a universal application. Another limitation is the detection of forest patches using remote sensing classification, the RF machine learning algorithm. For instance, a number of the patches from the study area would likely be as a result of the commission and omission errors measured. However, to reduce these uncertainties, different classification algorithms such as support vector machine [75] and artificial neural networks [76] can be tested and used depending on the algorithm’s performance. Additionally, more efforts should be made to reduce the effects of scale on classification results during satellite image processing.

## Methods

### Study area—profile of the Atewa range forest reserve and its surroundings

**Fig. 2:**
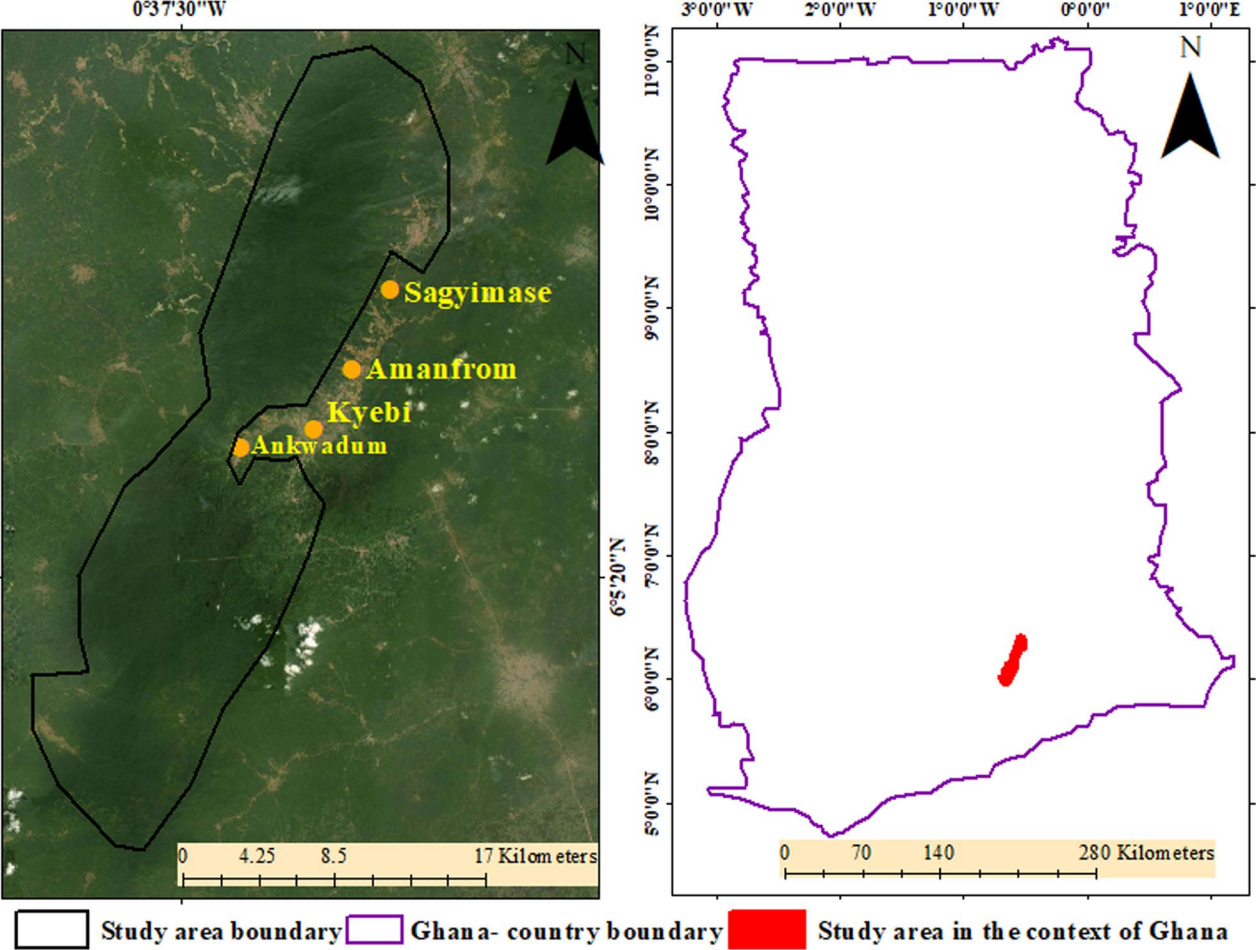
The study area location and some notable communities. The study area boundary and the Ghana country boundary were created by authors with the aid of Environmental Systems Research Institute ArcGIS 10 desktop basemaps

The study area, ARFR is in the East Akim Municipal area in the eastern region of Ghana (see Fig. 2). The forest reserve lies within a semi-deciduous forest area. The size of the forest reserve is 263 km^2^ [77]. Whereas the area is typically undulating in nature, some areas are as high as between 240 and 300 m above sea level. Additionally, the forest reserve can be found in the west semi-equatorial zone, an area that has two main rainy seasons. The first starts from May to June and the second from September to October [78]. The mean annual rainfall is between 125 and 175 mm. Following the rainy seasons is a dry season that starts in November and ends in late February. These weather dynamics facilitate plant growth and support most of the rain-fed farming activities at the fringes of the forest reserve.

The major economic activity in the East Akim municipality is farming as about 65% of the population are active farmers. Farmers engage in the planting of important cash crops such as Cocoa and Coffee as well as staple foodstuff, including cassava, maize, plantain, oil palm and banana [78]. Thus, encroachments by farmers at the fringes of the forest reserve are likely to reduce forest cover and the patches of forest. The study location has been designated as a reserve and a GSBA, but it is subject to unmanaged logging, uncontrolled hunting, artisanal gold and in recent times bauxite surface mining [77]. These activities are likely to result in forest cover loss and forest patch fragmentation and thus, threaten the rich biodiversity in the forest reserve. Some of the prominent communities or townships at the fringes of the ARFR are Kyebi, Amafrom, Sagyimase, and Ankwadum.

### Data

**Fig. 3:**
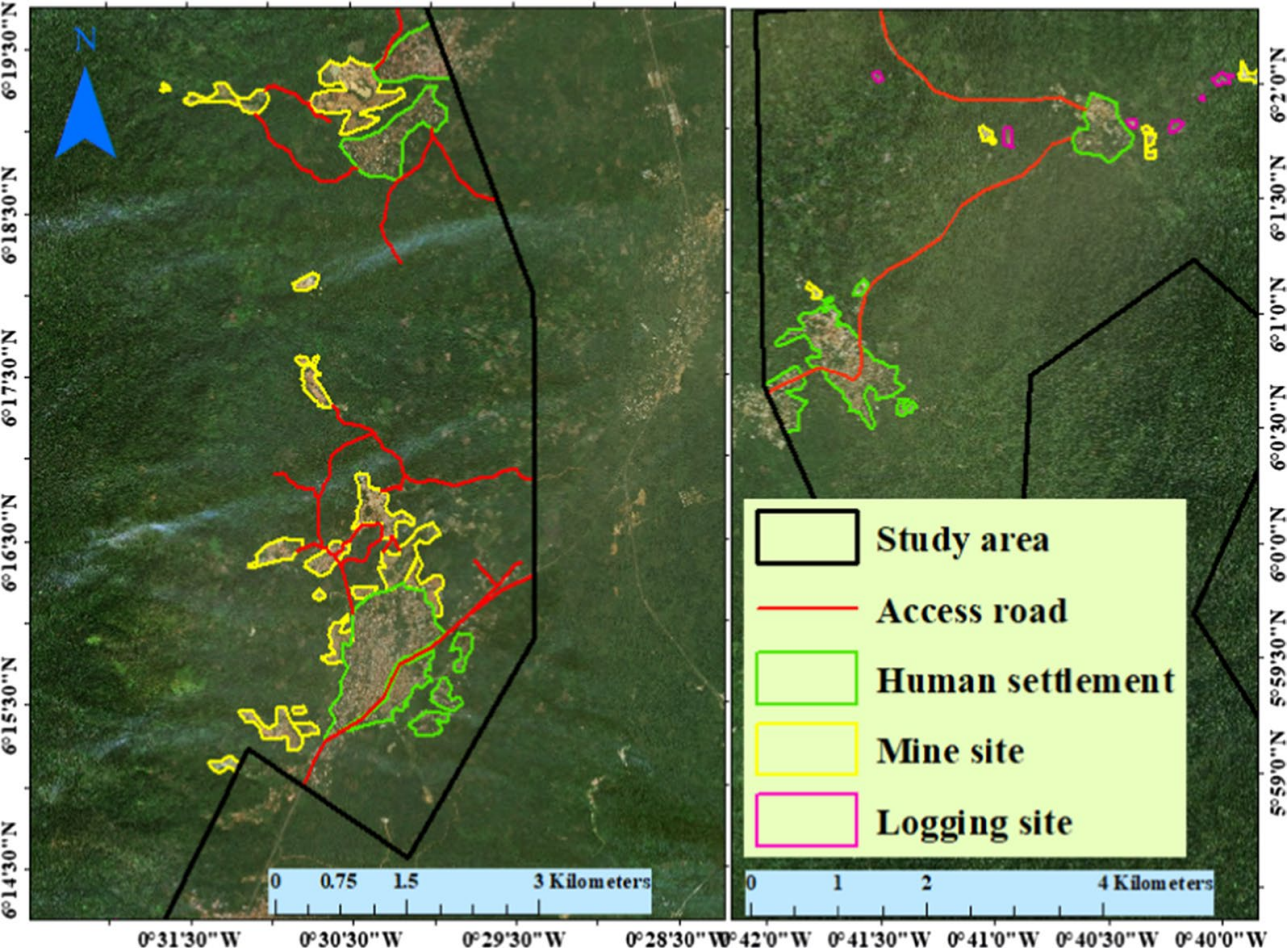
Examples of land use footprints (features) digitised from the high-resolution aerial images. This figure was created by authors with the aid of Google Earth Pro high-resolution images

The most recent footprints of land use were extracted from February 2020 high-resolution aerial images (1–1.5 m resolution) through digitising at a scale ranging between 1:5000 and 1:15,000 (see Fig. 3). These images were from the Environmental System Research Institute (ESRI) ArcGIS desktop and Google Earth Pro (GEP). These images were also used as ancillary information for identifying land classes during Landsat image classification. Furthermore, we collected 550 ground-truth reference samples from the high-resolution aerial images and used them for assessing the accuracy of the classified Landsat image.

We used a 30 m by 30 m resolution Landsat image from Landsat 8 Operational Land Imager/Thermal Infrared Sensor (OLI/TIRS). The Landsat image was acquired from the United States Geological Survey (USGS). The image is from WRS_PATH = 193, WRS_ROW = 56. The image used is a tier-one surface reflectance image which has been corrected geometrically and radiometrically. The image was captured by the Landsat satellite on 2020-02-03. We used a Landsat image from 2020 to be able to extract one of the most recent forest patches on the landscape of the ARFR.

### Landsat image classification and accuracy assessment

We composited bands 2–7 of the Landsat image and used Random Forest (RF) machine learning classification algorithm to classify the Landsat image into forest cover, agricultural land, developed land water. RF, a non-parametric classifier is noted for its high accuracy power in classifying objects and the ability to deal with non-normal and complex relationships among predictor variables [79–81]. RF uses an ensemble of decision trees predictors to produce repeated multiple classifications of the same data [82]. Predicted classes are combined from the decision trees using the maximum votes rule to generate the classes. In this study, training samples of forest (200), agricultural land (100), developed land (100), and water (100) were selected evenly from the Landsat image to train the RF classifier. The number of trees used in this study is 500 (by default) because according to Breiman and Cutler [83], there is no significant difference in the classification outcome for trees above the default selection.

**Fig. 4:**
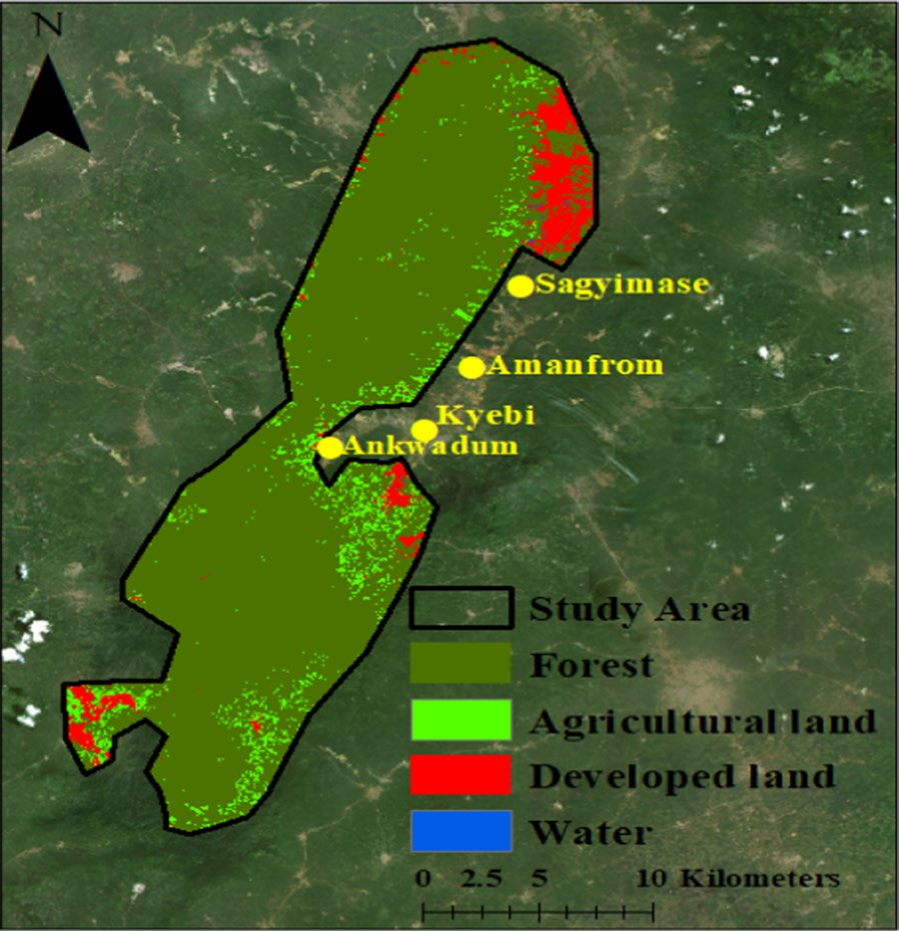
Land use land cover classes in the Atewa range forest reserve. This figure was created by authors using a Landsat image from the United States Geological Survey. Also, the high-resolution image in the figure is from Environmental Systems Research Institute’s ArcGIS 10 desktop basemap

The classes of land generated from the RF classification method are forest (mostly semi-deciduous trees), agricultural land (cropland, herbs and bushes), developed land (mine grounds, built-up area, roads and logging sites), and water (intermittent and permanent rivers, streams, lakes and other stagnant water) (see Fig. 4). The accuracy of these land categories classified from the Landsat image was assessed using the 550 randomly collected ground-truth samples. These ground-truth samples were pixels of land cover collected in February 2020 using a hand-held global positioning system (GPS) device. The GPS device used in the ground truth samples collection is the Garmin GPSMap 78 s, and the errors associated with the sample reference data collection range between 2 and 3 m. Despite difficulties we had in accessing the ARFR, we still made effort to collect pixels of land cover proportionate to the classes of land on the landscape of the study area while also making sure that the number of pixels collected for the rare land class (water) is not underrepresented. First, for the forest class, which is the largest land class (87.3%), we collected 186 pixels from the field. Second, for the agricultural land (second largest land class = 6.4%), we sampled 135 pixels, Third, for the developed land which is 6.2% of the total land, we sampled 128 pixels. Last, for the water class (0.1%), we sampled 101 pixels. We compared the land classes (see Fig. 4) predicted by the RF classification algorithm to the ground-truth samples collected from the field in the ARFR. The agreement and disagreement between the two sets of land classes are summarised in Appendix. An overall accuracy level of 96.36% was achieved in the Landsat image classification process (see Table 5 for details of the accuracy assessment results). Even though we achieved a high accuracy level (96.36%) with the reference data collected using a hand-held GPS, Congalton, Russell and Green [84] have noted that sample reference points collected with a GPS would still have some positional inaccuracies, including uncertainties about whether or not a reference point location acquired with a GPS is equivalent to a pixel. Positional inaccuracies are not avoidable, and thus, reference locations are biased due to vegetation and terrain interference with satellite signals needed to acquire accurate location data [85]. Furthermore, despite the GPS error of 2–3 m being low and within an acceptable range [84], the amount of error is likely to negatively affect the accuracy of the ground-truth samples and consequently impact the accuracy assessment of the classified Landsat image.

### Variables and analytical framework

#### Dependent variable

**Fig. 5:**
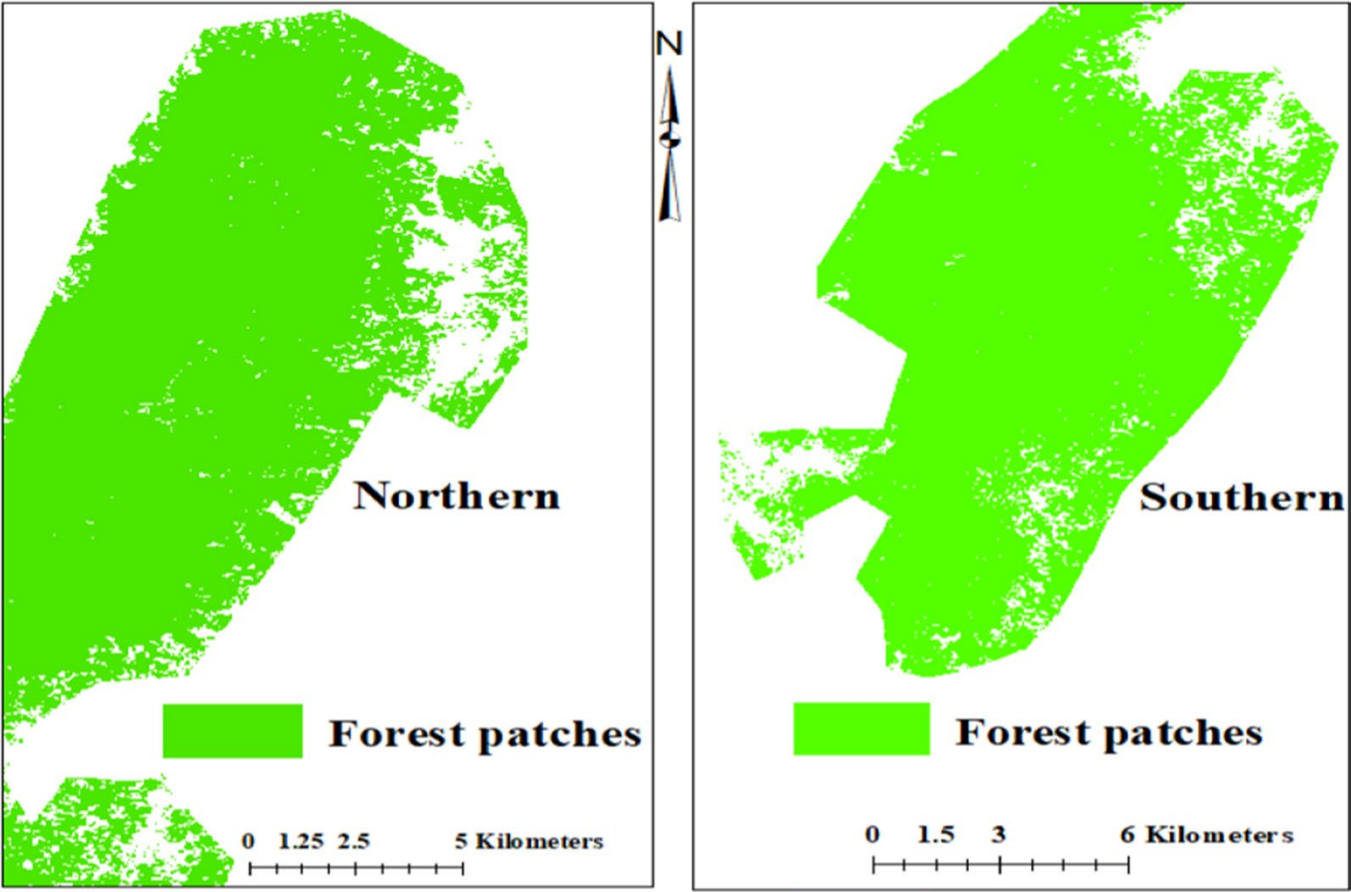
Patches of forest cover from the north–south divide of the Atewa Range Forest Reserve. This figure was created by authors using a Landsat image acquired from the United States Geological Survey

Forest cover patches were extracted from the classified Landsat image (see Fig. 5). Overall, a total of 865 patches of forest cover were extracted from the February 2020 classified Landsat image and all of them were used in the analysis. Whereas the largest forest patch is 315,004,261 m^2^ (31,500.43 ha), the smallest patch is 575 m^2^ (0.0575 ha). Also, the mean patch area of forest cover is 375431.87 m^2^ (37.54 ha). The majority of the forest patches (785) are less than the mean patch area, and this is an indication that most of these patches are not maximally aggregated or contiguous. Here, we select a dependent variable as the occurrence of patches that are less than the mean patch area (MPA) of 375,431.87 m^2^ (37.54 ha). Thus, we generate a binary of 1 and 0, where 1 means a forest patch is less than the MPA, and 0 means a forest patch is not less than the MPA (i.e., 1 = Yes, 0 = No).

#### Explanatory variables

The predictor or explanatory variables are nearness to agricultural land [1 = Yes, 0 = No], nearness to access roads [1 = Yes, 0 = No], nearness to mine sites [1 = Yes, 0 = No], nearness to logging sites [1 = Yes, 0 = No], and nearness to human settlement [1 = Yes, 0 = No], where nearness is determined by the likelihood of the forest patch being within or beyond 1 km distance from land use footprints or land use-induced land cover. As noted earlier, Haddad et al. [37] have indicated that 70% of the remaining forest is within 1 km from forest edge where human activities, including a variety of land uses, are taking place. A similar threshold distance within which human activities are concentrated in forest areas has been reported in the Brazilian Amazon [38]. However, much attention has not been given to the spatial relationship between the footprints of land uses and the most recent forest patch area. Thus, in this study, we selected this distance threshold based on previous study findings [37] to test the spatial relationship between the most recent forest patches and the footprint of land uses.

### Spatially-explicit modelling and measure of model fitness

In this study, we employed a spatially-explicit logistic regression equation to model the relationship between the most recent forest patches (dependent variable) and footprints of land uses (predictor variables) in the study area. Turner [44] has recommended the inclusion of multiple factors in a spatially-explicit landscape model in explaining aspects of landscape patterns. Here, we include multiple spatially-explicit land use factors in predicting forest patches in a GSBA. Based on the theoretical framework designed by Berkson [86] and Gujarati and Porter [87], we propose a full or saturated multivariate model to define the relationship between forest patches and footprints of land uses. The probability *P*_1_ of a forest patch area (PA) being less than the MPA of forest patches in the ARFR is given by

1 $${\text{PA = }}\left( {\frac{{{\text{P}}_{{1}} }}{{1 - {\text{P}}_{{1}} }}} \right){\text{ = X}}_{{\text{o}}} + {\text{X}}_{{1}} {\text{AL}}_{{{\text{1km}}}} + {\text{X}}_{2} {\text{AR}}_{{{\text{1km}}}} + {\text{X}}_{3} {\text{MS}}_{{{\text{1km}}}} + {\text{X}}_{4} LS_{{{\text{1km}}}} + {\text{X}}_{{5}} HS_{{{\text{1km}}}} + {\text{e}}$$

where *X*_0_ is the model intercept; *X*_1_,* X*_2_,* X*_3_,* X*_4_, and* X*_5_ are the regression model coefficients; *AL* = Agricultural Land, *AR* = access roads, *MS* = mine sites, *LS* = logging sites, *HS* = human settlement, and *e* is the stochastic error term.

The robustness of the model was tested as follows. First, we used the Hosmer and Lemeshow Test [88]. For the Hosmer and Lemeshow Test, the model is robust if *P* > 0.05. Second, we used the omnibus tests of model coefficients to do another test of model fitness. The omnibus test is a likelihood-ratio Chi-square test of the significant difference in the current model and the null (in this case, the intercept) model, the model with no independent variables. The significance value of less than 0.05 indicates that the current model outperforms the null model. This test is particularly important to test whether a regression model fit a dataset used in running the model [89]. Finally, we used the area under the curve (AUC) receiver operating characteristics (ROC), a well-known and widely-accepted measure used on measuring model performance [90]. The ROC value ranges between 0.5 and 1, where 0.5 and 1 show random and perfect model fit, respectively [43]. Previous studies that have reconciled methods from GIS, remote sensing and statistics have used the ROC to measure the robustness of models [90, 91].

## Appendix

See Table 5.

**Table 5:** Accuracy assessment of the classified land use land cover from the Atewa range forest reserve

		Reference data						
	Category of land	Forest	Agricultural land	Developed land	Water	Total truths	User accuracy (%)	Commission error (%)
**Predicted land class**	Forest	180	5	1	0	186	96.77	3.23
	Agricultural land	5	128	1	1	135	94.81	5.19
	Developed land	2	3	122	1	128	95.31	4.69
	Water	1	0	0	100	101	99.01	0.99
	Total	188	136	124	102	550		
	Producer accuracy (%)	95.74	94.12	98.39	98.04			
	Omission error (%)	4.26	5.88	1.61	1.96			
	Overall accuracy (%)	96.36						

## Abbreviations

ARFR: Atewa range Forest Reserve
AL: Agricultural Land
AR: Access Roads
AOR: Adjusted Odds Ratio
CI: Confidence Interval
ESRI: Environmental System Research Institute
GEP: Google Earth Pro
GIS: Geographic Information System
GSBA: Globally Significant Biodiversity Area
HS: Human Settlement
IBA: Important Bird Area
LS: Logging Site
RF: Random Forest
ROC: Receiver Operating Characteristics
MPA: Mean Patch Area
MS: Mine Site
OLI/TIRS: Operational Land Imager/Thermal Infrared Sensor
PA: Patch Area
SE: Standard Error
USGS: United States Geological Survey

## Data and material availability

The datasets used and/or analysed during the current study are available from the corresponding author on reasonable request.
